# Neglect, Abuse, and Adaptive Functioning: Food Security and Housing Stability as Protective Factors for Adolescents

**DOI:** 10.3390/children9030390

**Published:** 2022-03-10

**Authors:** Julia M. Kobulsky, Dalhee Yoon, Miguel T. Villodas, Brittany R. Schuler, Rachel Wildfeuer, José N. Reyes

**Affiliations:** 1 School of Social Work, College of Public Health, Temple University, Philadelphia, PA 19122, USA; brittany.schuler@temple.edu; 2 Department of Social Work, Binghamton University-State University of New York, Binghamton, NY 13902, USA; dyoon@binghamton.edu; 3 Department of Psychology, College of Sciences, San Diego State University, San Diego, CA 92121, USA; mvillodas@sdsu.edu; 4 Department of Sociology, Temple University, Philadelphia, PA 19122, USA; rachel.wildfeuer@temple.edu; 5 Department of Health Services Administration and Policy, College of Public Health, Temple University, Philadelphia, PA 19122, USA; jnrey3@temple.edu

**Keywords:** child maltreatment, adaptive functioning, protective factors, neglect, abuse, adolescence, resilience, healthcare, educational functioning, social functioning

## Abstract

This study addresses gaps in knowledge of protective factors that support adaptive functioning among maltreated adolescents. The sample included 1003 high-risk youths participating in the Longitudinal Studies of Child Abuse and Neglect (53% female, 56% Black, and 82% living in poverty). Adolescent neglect (Exposure to Risky Situations, Lack of Monitoring, Inattention to Basic Needs, Permitting Misbehavior, Lack of Support) and physical, sexual, and emotional abuse were self-reported at age 16. Age 18 adaptive functioning measures included healthcare receipt (medical, dental, and mental health), self-rated global health, high school graduation or enrollment, prosocial activities, peer relationships (Companionship, Conflict, Satisfaction, and Intimacy), and independent living skills. Previous childhood maltreatment, demographics, and earlier prosocial activities and peer relationships were controls. Structural equation modeling showed that adolescent neglect and abuse were associated with lower adaptive functioning. Multigroup models showed protective effects for food security on the relationships between sexual abuse and self-rated health and between Inadequate Monitoring and Companionship. Housing stability buffered relationships between Inadequate Support and high school graduation or enrollment and between Permitting Misbehavior and independent living skills. Findings imply the need for adolescent-focused prevention, including the promotion of food security and housing stability to support adaptive functioning in maltreated adolescents. However, notable mixed findings show the need for additional research.

## 1. Introduction

According to the National Survey of Children’s Exposure to Violence, more than a third (38%) of adolescents in the United States have experienced child maltreatment in their lifetime [1]. Similarly, a synthetic cohort lifetable analysis estimated that 37% of youths are reported to child protective services (CPS) for child maltreatment by 18 years of age [2]. The estimated lifetime economic burden of investigated child maltreatment during 2015 in the United States was USD 2 trillion [3]. In addition, many studies have identified that child maltreatment is associated with subsequent psychopathology, risk behaviors, and victimization [4,5,6]. Compounding the problem, unmet basic material needs for secure food and stable housing exist at disproportionately higher rates among families with maltreatment [7]. 

Though previous studies have articulated the consequences of maltreatment that occurs during childhood, limited research has focused on neglect and abuse that occurs during adolescence. In addition, prior studies have primarily been framed from a problem-focused or deficit perspective of child maltreatment, examining its effects on later maladaptation. Relatively little research has been framed from a resilience-focused perspective, considering the relationship between child maltreatment and adaptive functioning [8]. A resilience-focused perspective may provide clearer and more effective targets for prevention to advance the healthy development of maltreated youths. This study contributes to knowledge in this area by examining the relationships between neglect and abuse types during adolescence and subsequent adaptive functioning. In addition, it examines potential moderating effects of two potential key modifiable protective factors: food security and housing stability. 

### 1.1. Child Maltreatment and Adaptive Functioning

A resilience-focused perspective considers the capacity of an individual to adapt successfully to risks and challenges (e.g., child maltreatment) that could undermine adaptive functioning [9]. Adaptive functioning is the degree to which individuals perform well at social and interpersonal activities consistent with their development [10]. Given the key task of transitioning to independent adulthood in adolescence, key domains of adaptive functioning include health, high school completion, social connectedness (e.g., involvement in prosocial activities, positive relationships with friends), and independent living skills; these have been shown to predict health and well-being in emerging adulthood [11,12,13]. It is important to consider that resilience assumes adaptive functioning across multiple systems or domains, as an individual could be functioning adaptively in one domain (e.g., social connectedness) and maladaptively in another (e.g., high school completion) [9]. Thus, it is important to understand factors that can promote adolescent adaptive functioning across a variety of domains. 

Among other factors, a substantial body of research has documented that child maltreatment is inversely associated with adaptive functioning [8,14], including health-related life quality [15], academic performance [16], and social connectedness [17,18]. In a probability sample of Vietnamese adolescents (*N* = 1851), Tran et al. found that self-reports of lifetime physical and sexual abuse were associated with lower quality of physical health; however, emotional abuse was associated with better academic performance [19]. Alink et al. found that children (*M*_age_ = 7.6) with CPS-reports of maltreatment struggled to develop social functioning relative to non-maltreated children (i.e., without CPS reports) [20]. Lower levels of social functioning were related to lower morning cortisol levels one year later, which is a physiological indicator of stress response. Oshri et al. examined the growth patterns of social skills among adolescents reported to CPS for maltreatment (*N* = 1179); maltreated adolescents who had higher or increased levels of social skills (approximately 30% of the sample) reported better physical health, higher independent living skills, and higher grades [18]. 

Among different types of maltreatment, neglect specifically has been negatively associated with adaptive functioning, such as academic performance and peer relations [21,22,23]. An investigation on a rural Chinese sample (*N* = 2397) found that diverse dimensions of neglect (physical, educational, and medical) were inversely associated with social living ability (prosocial activities, social and educational functioning, communication, independent living, and self-management) [24]. 

### 1.2. Child Maltreatment during Adolescence 

Adolescence is the developmental period spanning childhood to adulthood during which multiple social and physical transitions take place [25]. Given neurological, cognitive, and social changes that occur during adolescence, it may be a sensitive period for child maltreatment exposure [26]. However, limited studies have focused on maltreatment specifically during adolescence [4,16,27]. An even smaller subset has included developmentally sensitive conceptualizations of maltreatment [4]. For example, neglect would be differently interpreted depending on development: leaving an infant or young child at home unsupervised is considered neglect, whereas doing so for an adolescent usually is not [28]. Finally, the limited existing research with developmentally specific measures of adolescent maltreatment has been framed from a deficit perspective, focusing on health risk outcomes [4]. This does little to enlighten the effective promotion of adaptive functioning during adolescence. 

### 1.3. Food Security and Housing Stability as Potential Protective Factors 

Research that can identify malleable factors to promote adaptive functioning in the face of child maltreatment is highly relevant to intervention. Most research and existing maltreatment interventions to date have targeted parent and parent–child relationship factors, such as parenting knowledge, behavior, and parental health [8,29]. Given the well-established relationship between child maltreatment and poverty, however, increasing calls have been made to further address the material needs of families at risk for maltreatment [30]. This includes interventions to assure that children’s basic needs for shelter (i.e., housing stability) and nutrition (i.e., food security) are met to hopefully prevent maltreatment and mitigate its deleterious effects. Of note, poverty and parental neglect are related but distinct factors that may both jeopardize housing and nutrition. 

Food security and housing stability are basic needs that are integral to daily functioning [31,32]. Consistent with family stress theory, the absence of housing stability and food security can seriously strain the family system, compromising child adjustment [33,34]. Preoccupation and stress within the family system around not having enough food or stable shelter and physiological effects of their absence, such as hunger, fatigue, irritability, and difficulty concentrating [31,32], could compromise adaptive functioning among maltreated adolescents. Thus, the presence of food security and housing stability may promote resilience in children and adolescents in the face of maltreatment through their stabilizing effects on family systems, including their contribution to basic physiological needs, by promoting attendance to non-emergency health, educational and social needs, and skill development. 

Empirical work supports food security and housing stability as potential moderators for the effects of child maltreatment on adolescent adaptive functioning. Inverse relationships between child maltreatment and housing stability and, to a lesser extent, food security, have been established [7,35]. For example, a systematic review of 21 articles found that housing instability, including homelessness, eviction, and multiple moves, is associated with caregiver-reported, child self-reported, and CPS indications of maltreatment [35]. Similarly, a study using a large United States sample found an inverse relationship between food security and child maltreatment [36]. 

DuMont et al. found that children who had experienced maltreatment were more likely to be successful in at least four of five domains of functioning, including graduating from high school, mental health, fewer substance use problems, fewer arrests, and less self-reported violence, if they grew up with both parents until age 18 or remained in their first out-of-home child welfare placement for more than 10 years [37]. Housing stability has been linked to children’s educational attainment and cognition/learning, healthcare receipt, health, and wellbeing [38]; this may buffer the negative effects of maltreatment. Similarly, consistent access to adequate, nutritious food has been linked to better educational, social, and health outcomes [31,39], potentially mitigating the effects of maltreatment during adolescence.

### 1.4. The Current Study 

The overarching objective of this study is to address gaps in knowledge on protective moderating factors—food security and housing stability—to inform interventions that will support adaptive functioning among maltreated adolescents. Using a developmentally sensitive, multidimensional measure of adolescent neglect with known psychometric properties [40], this study examines the effects of adolescent neglect and abuse types on adolescent adaptive functioning in the high-risk Longitudinal Studies of Child Abuse and Neglect (LONGSCAN) sample. Guided by resilience theory [9], we examine multiple domains of adaptive functioning, including health (healthcare receipt and self-rated global health), high school graduation or enrollment, social connectedness (prosocial activities and peer relationships), and independent living skills. We address three research questions. First, to what extent do adolescent neglect and abuse types at age 16 relate to later adolescent adaptive functioning at age 18, above and beyond childhood maltreatment, poverty, and age 16-year prosocial activities and peer relationships? We hypothesized that adolescent neglect and abuse types would be inversely associated with all domains of adaptive functioning. Second, does food security moderate the relationships between adolescent neglect and abuse types and adaptive functioning? We hypothesized that food security would play a protective role, such that associations between adolescent neglect and abuse types and adaptive functioning would be weaker for individuals with food security than those with food insecurity. Third, does housing stability buffer the relationships between adolescent abuse and neglect types and adaptive functioning? We hypothesized that housing stability would play a protective role in the same manner as food security. 

## 2. Method

### 2.1. Sample and Procedures 

The study sample was derived from LONGSCAN, a multi-site longitudinal cohort study following children and adolescents at risk for maltreatment from age 4 to age 18 in the United States [41]. Data collection was conducted every two years beginning in 1991 when children were 4 years old and ending in 2012, when the 18-year data collection was completed. Parent–child dyads were interviewed at years 4–16 and children were interviewed at 18 years. Except for interviews at age 10, which were conducted by phone, data were collected via in-person interviews, with sensitive information collected by Audio Computer-Assisted Self Interviews. In addition, relatively brief telephone interviews with parents were conducted the years between biannual interviews (i.e., the odd-numbered child ages) to facilitate a more complete picture of the family environment and the children’s development. 

Each of the five sites used the same data collection protocols and measures but different sampling strategies. Sampling frames included children investigated by CPS (northwestern and southwestern sites), children with risk factors for maltreatment (poverty, etc.) served by pediatric clinics (eastern site) and in public health tracking systems (southern site), and children reported to CPS or in high-risk groups that were matched on neighborhood, race/ethnicity, and SES (midwestern site). Adolescents in the analytic sample of *N* = 1003 are a subset of the original *N* = 1354 study participants and were included if they completed the age 16-year and/or the 18-year study measures and had valid data on the hypothesized moderators (i.e., food security, housing stability). Black youths were overrepresented in the analytic sample (56%) relative to those who were excluded (45%), χ^2^(1) = 13.485, *p* < 0.001. White youths were underrepresented (24% analytic sample vs. 33% excluded), χ^2^(1) = 10.033, *p* < 0.001. No other differences were found. This study involved secondary data analysis of deidentified data obtained from the National Data Archive on Child Abuse and Neglect; it was thus determined to be non-human subjects research by Temple University’s institutional review board.

### 2.2. Measures

#### 2.2.1. 16-Year Adolescent Neglect

The Mid-adolescent Neglect Scale, a LONGSCAN-developed instrument, was administered to youths at 16 years of age to assess past-year experiences of parental neglect. Youths responded on a 4-point Likert response scale (strongly disagree, disagree, agree, strongly agree). The scale has five confirmed dimensions of neglect: Inadequate Monitoring (e.g., “*wanted to know where I was if not at home*”; α = 0.81*),* Inattention to Basic Needs (e.g., “*made sure I had a safe place to be when I was not at school*”; α = 0.93*),* Permitting Misbehavior (e.g., “*if I had wanted to smoke cigarettes, my parents would have been upset*”; α = 0.78), Exposure to Risky Situations (e.g., “*were involved in loud fights that may have included hitting*; α = 0.81*),* and Inadequate Support (e.g., “*helped me when I had a problem*”; α = 0.91*).* These scales have demonstrated convergent validity [40]. 

#### 2.2.2. 16-Year Adolescent Abuse 

At age 16, the adolescents self-reported physical abuse, sexual abuse, and emotional abuse from the age of 12. The scales included 12 physical abuse (α = 0.67), 11 sexual abuse (girls α = 0.95, boys α = 0.89), and 12 emotional abuse (α = 0.81) yes/no items [42]. They were based on Barnett et al.’s conceptualizations of abuse [43]. Responses were yes (1) or no (0). Sum scores of items where abuse was indicated were calculated for each measure. 

#### 2.2.3. 18-Year Adaptive Functioning Variables 

Healthcare Receipt. Youths responded to three yes/no questions about the receipt of routine medical care (“*did you get a healthcare check-up?*”) dental care (*“did you get dental care, or a dental check-up?*”), and psychological counseling (*“did you get counseling or therapy for a psychological or emotional problem?*”) in the past 12 months. The psychological counseling question was combined with another question about whether these services were needed, since psychological counseling is not appropriate for everyone. The resulting variables indicated receiving (or not needing) routine medical, dental, or psychological care (1) and not receiving routine medical care, dental care, or needed psychological care (0). 

Self-rated Global Health. The youths self-reported their health to a single item: “compared to others, how would you say your health is?”. Responses were poor (1), fair (2), good (3), and excellent (4). This item is reliable and widely used [44].

Independent Living Skills. Independent living skills were assessed by the Ansell Casey Life Skills Assessment, Ages 11–18, Short Form. This 20-item scale measures adolescents’ practical life skills in 5 domains: money management, work-study skills, self-care, daily living skills, and social development. The total raw score was used, which represents the percentage of overall possible mastery (α = 0.90). Psychometric studies on the instrument have demonstrated internal consistency and test–retest reliability [45,46] with other work, demonstrating criterion validity [47]. 

High School Graduation or Enrollment. Caregivers reported by phone whether or not (*yes/no*) the youth “graduated from high school or received a GED” and were currently enrolled in high school. These questions were merged into a dichotomous variable indicating high school graduation or enrollment (*yes* = 1, *no* = 0). 

Prosocial Activities. Youths responded to 11 yes/no questions about participation in sports, clubs, performing arts, scout troops, volunteer groups, religious or church groups or activities, an apartment, block, neighborhood, or community meeting, political or advocacy group meeting, political rally or march, and solidarity or ethnic support groups [42]. The items were summed for a total count of activities within the past year (α = 0.68). 

Peer Relationships. Adapted from Furman and Burhmester, LONGSCAN’s Network of Relationships inventory was used to measure the quality of peer relationships [48]. The scale has four dimensions with 3 items each: Companionship (e.g., *how much free time do you spend with [friends];* α = 0.75), Conflict (e.g., *how much do you disagree and quarrel with [friends];* α = 0.81), Satisfaction (e.g., *how happy are you with the way things are between you and [friends];* α = 0.85), and Intimacy (e.g., *how much do you tell everything to [friends];* α = 0.82). Each question was asked separately about the best female friend, best male friend, male friend who is not a brother or boyfriend, female friend who is not a sister or girlfriend, boyfriend, and girlfriend. Youths indicated levels of satisfaction, frequency etc. on 5-point Likert-type response scales. Mean scores for each dimension were used (potential range: 15). 

#### 2.2.4. Moderating Variables

Food Security. Food security was measured by caregiver reports to 8 items. At 12, 14, and 16 years of age, caregivers reported on whether or not (*yes* = 1/*no* = 0) in the past 30 days the household ran out of money to buy food or the following occurred because there was not enough money to buy food: the household relied on a limited number of food items, caregivers ate less food, caregivers cut their own meal sizes or skipped meals, children said that they were hungry, children ate less than caregivers felt they should, caregivers cut children’s meal sizes or children skipped meals, and children went to bed hungry. Once summed, a score of zero indicated food security (1) and a score of one or higher indicated food insecurity (0).

Housing Stability. Housing stability was derived from annual administrations of LONGSCAN’s Life Events Scale from 12–17 years. At each interview, the caregiver reported whether in the past year: the family moved, the child moved away from the family, the child “didn’t have a place to stay and spent some nights with friends or relatives?”, “the child didn’t have a place to stay and spent some nights at a shelter”, and whether the child/family was evicted from their home. Housing stability (1) was indicated by one or fewer family moves over the 6-year period and no indication of the other experiences of housing stability. Housing instability (0) was indicated by two or more moves with the family over the 6-year period or any indication of other housing instability experiences. 

#### 2.2.5. Control Variables 

Childhood Maltreatment. Experiences of childhood abuse and neglect from 0 to 12 years were measured by self-report assessments at 12 years of lifetime physical abuse (15 items; α = 0.65), sexual abuse (11 items; α = 0.81), and emotional abuse (18 items; α = 0.82), and CPS reports of neglect through age 12. To accommodate for loss of memory for early childhood maltreatment, we also included CPS reports of any physical, sexual, or emotional abuse from age 0 to 4 years. These various indicators were combined to create a single dichotomous variable for childhood maltreatment (1 = yes, 0 = no). 

16-year Adaptive Functioning. At 16 years, youths reported prosocial activities and peer relationships for measures parallel to those used for the 18-year outcome measures. These were used as control variables to assist in establishing temporal ordering.

Demographics. Sex (male = 0, female = 1) and race/ethnicity were reported by caregivers at baseline. Multiple responses were not allowed. Due to small distributions of Hispanic (6.8%), Native American (0.6%), Asian (0.3%), Mixed Race (11.8%) and Other (0.6%) categories, race/ethnicity was recoded as White (reference), Black (1), or Other (1). Poverty was ascertained by whether youths lived under the federal poverty level at ages 4, 6, 8, 12, 14, or 16 (indication at one or more timepoints was coded as poverty). These were based on caregiver reports of household income, the number of dependents living in the household, and the federal poverty limit for the year corresponding with data collection.

### 2.3. Analysis 

#### 2.3.1. Descriptive and Bivariate Analyses

Means and standard deviations were assessed for all continuous variables. For categorical and ordinal variables, frequency distributions were assessed (Table 1). Bivariate subgroup (i.e., food secure and insecure, housing stable and unstable) differences were assessed using t-tests and chi-squared tests. Bivariate correlations were examined to check for multicollinearity, using Allison’s criteria of *R*^2^ < 0.60 [49].

#### 2.3.2. Structural Equation Modeling 

Single Group. Using a previously specified measurement model for adolescent neglect (see Figure S1), we conducted structural equation modeling in the overall sample [4,40]. Structural equation modeling was selected for its ability to incorporate the adolescent neglect latent variable and multiple outcome variables into a single model, reduce measurement error, and easily apply advanced missing data techniques to reduce bias [50]. In a single model, the eleven outcome variables were regressed on the 5-factor measurement model for adolescent neglect, adolescent sexual abuse, physical abuse, and emotional abuse scores, and control variables (i.e., race/ethnicity, sex, poverty, and childhood maltreatment). In addition, 18-year measures of prosocial activities and peer relationships were regressed on the corresponding 16-year measures. Weighted least squared square means and variance adjusted (WLSMV) estimation was used. Independent living skills, peer relationships, and prosocial activities were treated as continuous; standardized betas are reported for these. All other variables were treated as categorical or ordered categorical. No interpretable effect size is provided by M*plus* for categorical and ordered categorical outcomes with WSLMV [51]; therefore, unstandardized betas are reported for these. Hu and Bentler’s fit criteria were applied: Root Mean Square Error of Approximation (RMSEA) < 0.06, Tucker Lewis Index (TLI) > 0.95, and Standardized Root Mean Squared Residual (SRMR) < 0.08 [52]. Pairwise present was used to manage item-level missingness.

Multigroup. Following analysis of the one-group structural equation model, we conducted multigroup analysis to assess moderated effects for housing stability and food security status. In order to rule out the possibility that any group differences in structural paths were due to measurement bias, measurement invariance analyses were first conducted to assess whether the configuration of the items (configural invariance) and the contribution of items to factors (metric invariance) in the 5-factor adolescent neglect measurement model were equivalent between groups [53]. Configural models were first run to simultaneously examine the measurement model across the subgroups with no constraints, examining the direction and significance of item loadings. Metric invariance was then tested to assess the group equivalence of the relationships between scale items and latent variables. This was accomplished by comparing the configural model to the subsequent model in which factor loadings were constrained to be equal across groups. Deteriorations in model fit, including a significant log likelihood ratio test and deterioration in RMSEA, TLI, and SRMR would indicate metric non-invariance [54]. 

Next, multiple group structural equation modeling analysis was conducted. Two sets of models determined whether (1) food security and (2) housing stability moderated relationships between adolescent neglect and abuse and subsequent adaptive functioning. Group differences were tested by labeling and creating difference terms for individual structural paths with the M*plus’* “Model Constraint” command (e.g., “path1_secure − path1_insecure = path1_difference”). M*plus* provides estimates for difference terms (i.e., moderated effects) and associated Wald tests. 

## 3. Results

### 3.1. Descriptive and Bivariate Statistics

In the overall sample, two thirds of the youths (65%) reported receiving dental care in the past year, making it the least frequently met healthcare need followed by regular medical check-ups (79%) and counseling/therapy (93%). Three quarters (76%) of youths perceived themselves to be in excellent or good health. Total scores indicated 81% mastery of independent living skills (range: 33–100). On average, the youths engaged in 2 out of 11 prosocial activities (range: 0–9). Four fifths (79%) of the youths had graduated from or were currently enrolled in high school. Half (49%, *n* = 486) were food secure and 62% (*n* = 618) had stable housing. 

Several statistically significant group differences were found at the bivariate level. Of note, more youths who were food secure had graduated from or were enrolled in high school (87%) compared to those experiencing food insecurity (73%), and they had lower conflict with friends at age 18 (*M* = 2.3 vs. 2.4). Significantly more youths with stable housing had graduated from or were enrolled in high school (84%) than those who had experienced housing instability (72%), and they had lower adolescent sexual abuse scores (0.18 vs. 0.38). 

### 3.2. Structural Equation Model—Single Group

Parameter estimates for the single group model are shown in Table 2. Model fit indices were within range (see Table 3). More Inadequate Monitoring was related to lower Companionship (β = −0.13, *SE =* 0.06, *p* = 0.037), Satisfaction (β = −0.13, *SE =* 0.06, *p* = 0.023), and Intimacy with friends (β = −0.15, *SE =* 0.06, *p* = 0.010), but higher self-rated health (B = 0.19, *SE* = 0.07, *p =* 0.008). Greater Inattention to Basic Needs was associated with a lower likelihood of receiving dental care in the past year (B = −0.61, *SE* = 0.24, *p* = 0.011*),* but higher independent living skills (β = 0.48, *SE =* 0.18, *p* = 0.006). Permitting Misbehavior was related to engagement in fewer prosocial activities (β = −0.13, *SE =* 0.06, *p* = 0.026). Greater Exposure to Risky Situations (β = −0.29, *SE* = 0.09, *p =* 0.001) and Inadequate Support (β = −0.40, *SE* = 0.12, *p =* 0.001) were related to lower independent living skills. Greater Inadequate Support was also related to higher conflict with friends (β = 0.29, *SE =* 0.12, *p* = 0.015). 

Physical abuse was associated with lower independent living skills (β = −0.12, *SE* = 0.04, *p =* 0.004). Emotional abuse was related to lower self-rated health (B = −0.06, *SE* = 0.03, *p* = 0.028) but higher independent living skills (β = 0.13, *SE* = 0.05, *p* = 0.014). Sexual abuse was associated with lower Companionship (β = −0.08, *SE =* 0.04, *p* = 0.038) and Intimacy (β = −0.11, *SE =* 0.05, *p* = 0.030).

### 3.3. Structural Equation Model—Food Security Multigroup Analysis

Measurement invariance testing for the food security groups revealed configurations within groups were as expected and there was no significant deterioration in fit between the configural and metric models (Table 3). Thus, structural analysis proceeded with this support for measurement invariance. Structural models for the individual outcomes were run separately due to non-convergence of the larger model. Problems with nonconvergence and larger standard errors associated with the small subgroup sizes were addressed by simplifying the race variable to Black or non-Black, and removing childhood maltreatment from the independent living model.

The multigroup analysis identified several significant group differences for the food secure and insecure groups (see underlined coefficients in Table 4). Sexual abuse was related to higher self-rated health in the food secure group, but not the food insecure group (Difference = 0.26, *SE* = 0.08, *p* = 0.001). Another moderated effect indicated a relationship between Inadequate Monitoring and lower Companionship in the food insecure group, but no relationship in the food secure group (Difference = −0.20, *SE* = 0.08, *p* = 0.013). Finally, sexual abuse was related to higher Conflict with friends, but only in the food secure group (Difference = −0.12, *SE* = 0.05, *p =* 0.014).

### 3.4. Structural Equation Model—Housing Stability Multigroup Analysis

Modifications were made to address errors in model identification at the measurement invariance testing stage for the housing stability groups: (1) item 20 was cut from the Inattention to Basic Needs factor and (2) an error covariance between items 1 and 2 was removed. As shown in Table 3, the log ratio test of model differences between the configural and metric models was significant. However, all other model fit indices remained within thresholds. Thus, analysis proceeded with this partial support for measurement invariance.

Several differences were also found for the housing stability groups (see underlined path coefficients in Table 5). More Inadequate Support was associated with a lower likelihood of graduating from or being enrolled in high school, but only in the unstable housing group (Difference = 1.65, *SE* = 0.57, *p* = 0.004). More Permitting Misbehavior was related to lower independent living skills only when housing was unstable, but not in the stable housing group (Difference = 3.77, *SE* = 1.77, *p =* 0.033). In addition, more Inadequate Monitoring was associated with a greater likelihood of high school graduation or enrollment when housing was unstable, but a lower likelihood of graduation or enrollment when housing was stable (Difference = 0.85, *SE* = 0.28, *p =* 0.002). Emotional abuse was unrelated to prosocial activities in the stable housing group but was related to higher prosocial activities in the unstable housing group (Difference = −0.25, *SE* = 0.10, *p =* 0.014). 

## 4. Discussion

The present study fills an important gap in the literature by examining the association between neglect and abuse during adolescence and later adaptive functioning, as well as factors that could mitigate this association. Overall, findings from this study show that neglect and abuse during adolescence impairs later adolescent adaptative functioning. We also found evidence that food security and housing stability have protective effects, mitigating many of these associations, though the directions for some effects were not as hypothesized. The results of this study provide important insights into the potential protective roles of food security and housing stability for promoting adaptive functioning among adolescents who have been maltreated. 

### 4.1. Healthcare Receipt and Perceived Health

Our findings extend prior research that has connected child maltreatment to poorer health [15,19] to adolescent neglect and abuse types. Poverty and Inattention to Basic Needs (e.g., arranging for healthcare needs, ensuring appropriate clothing and shelter) were independently associated with lower odds of receiving dental care, consistent with research showing effects of neglect above and beyond poverty, as well as the conceptual distinction of poverty and basic needs neglect [55]. It was surprising that no adolescent neglect or abuse types were associated with receiving routine medical care or needed psychological counseling. It is important to note, however, that less than 8% reported unmet psychological healthcare needs and receiving needed psychological healthcare is confounded with having mental health challenges. 

Though emotional abuse was related to lower self-rated health, sexual abuse was associated with better self-rated health among youths who experienced food security but not food insecurity. The latter finding may indicate protective effects for food security against the putative negative health impacts of sexual abuse [56]. However, it was surprising that the negative association between sexual abuse and self-rated health was not significant among adolescents who experienced food insecurity. There were also counterintuitive findings between Inadequate Monitoring and *higher* self-rated health. Moderation analyses revealed that this association was only significant among youths who experienced food security; this is consistent with the hypothesized protection of food security. Nonetheless this finding was surprising. It is possible that the temporal ordering of the relationship was reversed, such that greater parental monitoring is observed in response to poorer adolescent health, particularly among families who are food secure. This could also explain, in part, unexpected findings in the multigroup analysis showing that Inadequate Monitoring was linked to higher high school graduation when housing is unstable, but lower graduation/enrollment when housing is stable. Of note, however, this same construct was found to predict *lower* substance use in a previously published study [4]. The Inadequate Monitoring scale contains only three items of parental knowledge and interest in children’s activities; unexpected findings therefore may suggest a lack of validity for the complex monitoring construct. 

### 4.2. High School Graduation or Enrollment

Past research has shown a detrimental effect of child and adolescent maltreatment on academic outcomes [16,18,22,23]. Although adolescent maltreatment did not predict high school graduation or enrollment in the single group analysis, in the multiple group analysis, Inadequate Support was associated with lower high school graduation or enrollment for adolescents who experienced housing instability. However, this association was nonsignificant for adolescents who experienced housing stability. Consistent with our hypotheses, this may suggest a protective effect of housing stability against the negative effects of poor parental support on high school graduation or enrollment and extends evidence on the protective effects for housing stability [38]. 

### 4.3. Social Connectedness

Findings generally corroborate past research demonstrating relationships between child maltreatment and social connectedness [17,18,20,24] and extend these findings to neglect during adolescence. In line with study hypotheses, Permitting Misbehavior was associated with less involvement in prosocial activities. Emotional abuse was associated with more involvement in prosocial activities among adolescents who experienced unstable housing. It is possible that adolescents experiencing the stresses of emotional abuse and unstable housing seek out positive adult and peer support and attention through engagement in prosocial activities. 

Regarding peer relationships, Inadequate Monitoring was related to less Companionship, Satisfaction, and Intimacy; sexual abuse was additionally related to less Companionship and Intimacy, and Inadequate Support was associated with more Conflict. Surprisingly though, sexual abuse was related to higher conflict with friends only when food was secure. This finding may indicate that food security promotes stability in dysfunctional peer relationships. 

Consistent with our hypothesis that food security would mitigate the effects of adolescent maltreatment on peer relationships, moderation analyses revealed that the negative association between Inadequate Monitoring and Companionship was only significant among youths who experienced food insecurity. However, inconsistent with our hypotheses, Inadequate Monitoring was only associated with less involvement in prosocial activities among adolescents who experienced housing stability. These findings should be interpreted with caution though given the previously mentioned concerns about the validity of this construct. 

### 4.4. Independent Living Skills

In line with past research [18,24], Exposure to Risky Situations, Inadequate Support and physical abuse were associated with poorer independent living skills. Moderation showed that Permitting Misbehavior was also associated with poorer independent living skills among youths who experienced unstable housing, a potential additional cost of permissiveness in an unstable environment. Emotional abuse and Inattention to Basic Needs, on the other hand, were associated with better independent living skills. Though unexpected, these findings have some precedent in the literature [19]. Neglected children have been found to have stronger adaptive functioning (problem solving, abstraction, and planning) than non-maltreated children in some research [21]. This may be related to adolescents being forced to take on adult responsibilities for their own survival. 

### 4.5. Limitations

This paper has several limitations. The high-risk nature of the sample limits generalizability; for example, we may have found more or different moderated effects for food security and housing stability in a sample with lower poverty. Second, although we used a longitudinal design, repeated measures of most dependent variables were not available, leaving questions about temporal sequencing. Third, race and ethnicity were not measured separately in LONGSCAN, and were reported by caregivers at birth versus being reported by the youths themselves. In addition, because of low frequencies in individuals who were not Black or White individuals, our analysis combined heterogeneous subgroups. Fourth, the childhood maltreatment variable may not be sensitive enough to detect unique effects (e.g., subtype differences). Fifth, we relied on self-reported measures of adolescent maltreatment; although these have greater sensitivity than CPS reports, they are subject to self-report bias [57]. Last, given model complexity and limitations in LONGSCAN measures of social interventions, we were unable to adjust for their potential influence.

### 4.6. Implications 

The results of this study suggest several implications for research and policy. Findings indicate the importance of maltreatment prevention for adolescents. This is currently a notable gap in the literature and real-world practice, where prevention has overwhelmingly focused on early childhood. Evidence provided by this study suggests that concerted efforts to prevent maltreatment from occurring (or recurring) during the adolescent years may foster a strong foundation for independent adulthood.

Findings further suggest the importance of providing strength-based services to enhance the resilience of adolescents who experience neglect and abuse. Specifically, they infer that strength-based services to buffer impacts of earlier maltreatment on adaptive functioning over time might focus on supporting the basic, material needs of adolescents for food security and housing stability. These may include enhancing outreach services that support food and housing needs. For example, facilitating enrollment in government support programs such as the Supplemental Nutrition Assistance Program (SNAP) and housing voucher programs, as well as programs such as those to enhance overall family income (e.g., Temporary Aid for Needy Families, Earned Income Tax Credit) may support adaptive functioning in adolescents. However, for families living with high levels of poverty, public support programs are often not enough to fill the financial hardship gaps and many families in need may not consistently meet eligibility requirements. For example, waitlists for housing assistance are several years-long [58]. In our study sample, 52% of families reported receiving food stamps, 83% reduced/free lunches, and 27% housing subsidies, but far fewer (20%, 48%, and 12%, respectively) received these benefits consistently over the 12–18-year time period. Receipt of those benefits did not equate with food security and housing stability; therefore, greater efforts are needed. These might include advocacy to expand safety net programs and otherwise make safe housing and nutritious food more affordable and available as well as poverty reduction efforts, such as raising the minimum wage. Greater integration between child welfare and safety net systems is needed [30]. For example, some child welfare jurisdictions have partnered with housing authorities and local landlords to connect families with Housing Choice Vouchers to apartments [35].

Although our analysis demonstrates some promise for housing stability and food security to help promote adaptive functioning among maltreated youths, notable mixed findings suggest these approaches are not a panacea. Nor are they a replacement for programs focused on parent or child health. In addition, further research is needed to clarify many of the relationships examined in this analysis, to examine additional protective and risk factors that may moderate these relationships, and mediated pathways. These might include studies considering the role of housing quality as well as stability and in-depth analyses of individual adaptive functioning domains and childhood maltreatment. Future research is also needed to replicate these findings, including in light of pandemic-related changes to safety net programs, food costs, and housing markets.

## 5. Conclusions 

This study demonstrates effects of neglect and abuse during adolescence on subsequent adaptive functioning, using a developmentally specific, multidimensional measure of neglect and controlling for prior childhood maltreatment. Findings indicate that neglect and abuse during adolescence impairs later adolescent adaptative functioning. Findings also suggest that food security and housing stability are potential protective factors that may mitigate the deleterious effects of maltreatment on adaptive functioning. 

## Figures and Tables

**Table 1:** Frequencies of Study Variables in Overall Sample, Food Security Groups, and Housing Stability Groups.

	Total Sample	Food Secure *N* = 486	Food Insecure *N* = 517		Housing Stable *N* = 618	Housing Unstable *N* = 385	
	*N* = 1003						
	N (%)/M(SD)	N (%)/M(SD)	N (%)/M(SD)	Statistical Test	N (%)/M(SD)	N (%)/M(SD)	Statistical Test
*Adaptive Functioning Outcomes at 18 years*							
Received medical check-up	671 (79.2)	328 (78.5)	343 (80.0)	χ^2^ (1, N = 847) = 0.28	422 (79.3)	249 (79.0)	χ^2^ (1, N = 847) = 0.01
Received dental care	553 (65.2)	279 (66.7)	274 (63.7)	χ^2^ (1, N = 848) = 0.86	349 (65.5)	204 (64.8)	χ^2^ (1, N = 848) = 0.05
Received/did not need counseling/therapy	782 (92.5)	390 (93.5)	392 (91.6)	χ^2^ (1, N = 845) = 1.15	491 (92.6)	291 (92.4)	χ^2^ (1, N = 845) = 0.02
Self-rated health							
Excellent	263 (30.9)	137 (32.6)	126 (29.2)	χ^2^ (1, N = 851) = 1.83	168 (31.5)	95 (30.0)	χ^2^ (1, N = 851) = 0.28
Good	384 (45.1)	188 (44.8)	196 (45.5)		239 (44.8)	145 (45.7)	
Fair	184 (21.6)	87 (20.7)	97 (22.5)		114 (21.3)	70 (22.1)	
Poor	20 (2.4)	8 (1.9)	12 (2.8)		12 (2.4)	7 (2.2)	
Graduated or in high school	466 (79.1)	230 (86.5)	236 (73.1)	χ^2^ (1, N = 589) = 15.86 ***	298 (83.7)	168 (72.1)	χ^2^ (1, N = 589) = 11.48 ***
Prosocial activities	1.9 (1.9)	1.9 (1.8)	2.0 (2.1)	*t* (828.98) = 0.317	2.02 (1.9)	1.79 (1.9)	*t* (838) = −1.61
Companionship	3.4 (0.68)	3.5 (0.6)	3.5 (0.7)	*t* (838) = 0.335	3.49 (0.6)	3.45 (0.7)	*t* (838) = −0.91
Conflict	2.3 (0.73)	2.3 (0.7)	2.4 (0.7)	*t* (838) = 2.18 *	2.31 (0.8)	2.34 (0.7)	*t* (715.19) = 0.56
Satisfaction with Friends	4.1 (0.59)	4.1 (0.6)	4.1 (0.6)	*t* (838) = −0.94	4.13 (0.6)	4.09 (0.6)	*t* (838) = −0.96
Intimacy	3.4 (0.85)	3.5 (0.8)	3.5 (0.8)	*t* (838) = 0.857	3.54 (0.8)	3.50 (0.8)	*t* (838) = −0.99
Total independent living skills	80.9 (12.7)	81.1 (12.4)	80.6 (12.9)	*t* (829) = −0.86	81.0 (12.7)	80.7 (12.6)	*t* (829) = −0.36
*Adolescent Abuse (12–16 years)*							
Physical abuse	0.4 (0.9)	0.3 (0.9)	0.4 (1.0)	*t* (723) = 1.13	0.31 (0.8)	0.44 (1.1)	*t* (452.52) = 1.67
Emotional abuse	0.8 (1.6)	0.7 (1.5)	0.9 (1.7)	*t* (707.96) = 1.53	0.73 (1.4)	0.96 (2.0)	*t* (433.31) = 1.72
Sexual abuse	0.3 (1.2)	0.2 (0.9)	0.3 (1.4)	*t* (666.92) = 1.48	0.18 (0.9)	0.38 (1.5)	*t* (399.29) = 1.98 *
*Control Variables*							
Childhood maltreatment (0–12 years)	760 (76.2)	357 (74.1)	403 (78.3)	χ^2^ (1, N = 997) = 2.41	432 (70.2)	328 (85.9)	χ^2^ (1, N = 997) = 31.73 ***
Female	526 (52.8)	271 (56.2)	255 (49.5)	χ^2^ (1, N = 997) = 4.50 *	327 (53.2)	199 (52.1)	χ^2^ (1, N = 997) = 0.11
White	239 (24.0)	121 (25.1)	118 (22.9)	χ^2^ (1, N = 997) = 0.66	143 (23.3)	96 (25.1)	χ^2^ (1, N = 997) = 0.46
Black	561 (56.3)	254 (52.7)	307 (59.6)	χ^2^ (1, N = 997) = 4.84 *	363 (59.0)	198 (51.8)	χ^2^ (1, N = 997) = 4.95 *
Other	197 (19.8)	107 (22.2)	90 (17.5)	χ^2^ (1, N = 997) = 3.50	109 (17.7)	88 (23.0)	χ^2^ (1, N = 997) = 4.20 *
Poverty	820 (82.2)	363 (75.3)	457 (88.7)	χ^2^ (1, N = 997) = 30.74 ***	484 (78.7)	336 (88.0)	χ^2^ (1, N = 997) = 13.84 ***
Prosocial activities (16 years)	2.1 (1.5)	2.2 (1.6)	2.0 (1.5)	*t* (782.60) = −2.12 *	2.19 (1.5)	1.86 (1.5)	*t* (816) = −3.06 **
Companionship (16 years)	3.4 (0.7)	3.4 (0.7)	3.4 (0.7)	*t* (815) = 0.271	3.40 (0.7)	3.39 (0.7)	*t* (815) = −0.226
Conflict (16 years)	2.2 (0.7)	2.2 (0.6)	2.3 (0.7)	*t* (815) = 1.44	2.20 (0.7)	2.27 (0.7)	*t* (815) = 1.39
Satisfaction with friends (16 years)	4.1 (0.6)	4.1 (0.6)	4.1 (0.6)	*t* (814) = −0.58	4.07 (0.6)	4.07 (0.6)	*t* (814) = 0.12
Intimacy (16 years)	3.4 (0.9)	3.4 (0.9)	3.4 (0.9)	*t* (815) = −0.539	3.35 (0.9)	3.42 (0.8)	*t* (815) = 1.12

Notes: M = mean, SD = standard deviation. Statistical tests are chi-squared tests of independence for categorical variables and independent sample *t*-tests for continuous variables. * *p* < 0.05, ** *p <* 0.01, *** *p <* 0.001.

**Table 2:** Structural Equation Model Predicting Functional Adaptations (*N* = 1003).

	Received Medical Check-Up		Received Dental Care		Received Needed Counseling/Therapy		Self-Rated Health		Graduated or in High School			
	B (*SE*)	*p*	B (*SE*)	*p*	B (*SE*)	*p*	B (*SE*)	*p*	B (*SE*)		*p*	
*Adolescent Neglect*												
Inadequate Monitoring	0.12 (0.09)	0.204	0.07 (0.09)	0.397	−0.19 (0.12)	0.121	0.19 (0.07) **	0.008	−0.03 (0.11)		0.795	
Inattention to Basic Needs	−0.23 (0.26)	0.382	−0.61 (0.24) *	0.011	0.09 (0.40)	0.824	−0.32 (0.19)	0.098	−0.29 (0.32)		0.373	
Permitting Misbehavior	0.07 (0.10)	0.469	0.08 (0.09)	0.341	0.10 (0.12)	0.403	0.11 (0.07)	0.115	−0.20 (0.11)		0.075	
Exposure to Risky Situation	0.08 (0.14)	0.587	0.15 (0.13)	0.242	−0.14 (0.20)	0.481	0.17 (0.10)	0.097	0.20 (0.16)		0.207	
Inadequate Support	−0.02 (0.18)	0.925	0.26 (0.17)	0.123	−0.32 (0.27)	0.235	−0.22 (0.14)	0.121	0.17 (0.23)		0.473	
*Adolescent Abuse*												
Physical Abuse	0.07 (0.08)	0.423	0.08 (0.07)	0.272	−0.13 (0.11)	0.237	0.03 (0.05)	0.612	0.13 (0.10)		0.167	
Emotional Abuse	0.00 (0.05)	0.966	−0.01 (0.04)	0.823	0.00 (0.06)	0.964	−0.06 (0.03) *	0.028	−0.06 (0.06)		0.316	
Sexual Abuse	0.01 (0.05)	0.773	0.00 (0.05)	0.946	0.14 (0.27)	0.609	0.01 (0.04)	0.760	−0.04 (0.06)		0.457	
*Control Variables*												
Child Maltreatment (0–12 years)	−0.06 (0.12)	0.601	−0.18 (0.11)	0.116	−0.19 (0.17)	0.255	−0.04 (0.09)	0.648	−0.28 (0.13) *		0.041	
Female	0.41 (0.10) ***	0.000	0.18 (0.09)	0.058	−0.20 (0.14)	0.148	−0.36 (0.08) ***	0.000	0.18 (0.12)		0.142	
Black (ref.: White)	0.18 (0.14)	0.204	0.08 (0.13)	0.562	−0.22 (0.18)	0.206	0.21 (0.10) *	0.032	0.44 (0.18) *		0.015	
Other (ref.: White)	−0.04 (0.15)	0.803	0.16 (0.15)	0.288	−0.35 (0.19)	0.070	0.21 (0.11)	0.068	0.50 (0.22) *		0.024	
Poverty	0.02 (0.14)	0.918	−0.43 (0.13) **	0.001	0.05 (0.18)	0.722	0.03 (0.11)	0.769	−1.01 (0.23) ***		0.000	
Outcome (16 years)												
	**Prosocial Activities**		**Companionship**		**Conflict**		**Satisfaction**		**Intimacy**		**Total Independent Living Skills**	
	**β (SE)**	***p***	**β (SE)**	***p***	**β (SE)**	***p***	**β (SE)**	***p***	**β (SE)**	***p***	**β (SE)**	***p***
*Adolescent Neglect*												
Inadequate Monitoring	−0.03 (0.06)	0.567	−0.13 (0.06) *	0.037	−0.09 (0.06)	0.097	−0.13 (0.06) *	0.023	−0.15 (0.06) *	0.010	−0.07 (0.06)	0.253
Inattention to Basic Needs	−0.08 (0.18)	0.644	−0.15 (0.18)	0.401	−0.17 (0.18)	0.361	−0.25 (0.18)	0.176	−0.04 (0.17)	0.799	0.48 (0.18) **	0.006
Permitting Misbehavior	−0.13 (0.06) *	0.026	0.03 (0.07)	0.681	0.05 (0.07)	0.470	0.03 (0.06)	0.635	−0.04 (0.06)	0.525	−0.08 (0.07)	0.248
Exposure to Risky Situation	0.05 (0.09)	0.558	0.01 (0.09)	0.915	0.05 (0.10)	0.646	0.08 (0.10)	0.452	0.05 (0.10)	0.600	−0.29 (0.09) **	0.001
Inadequate Support	0.05 (0.12)	0.669	0.10 (0.12)	0.415	0.29 (0.12) *	0.015	0.13 (0.12)	0.276	0.07 (0.11)	0.570	−0.40 (0.12) **	0.001
*Adolescent Abuse*												
Physical Abuse	−0.01 (0.04)	0.788	0.04 (0.04)	0.341	0.02 (0.04)	0.559	0.08 (0.04)	0.056	0.04 (0.05)	0.390	−0.12 (0.04) **	0.004
Emotional Abuse	0.05 (0.04)	276	−0.04 (0.05)	0.416	0.00 (0.05)	0.995	−0.07 (0.04)	0.098	−0.01 (0.05)	0.792	0.13 (0.05) *	0.014
Sexual Abuse	−0.02 (0.05)	0.751	−0.08 (0.04) *	0.038	−0.02 (0.03)	0.516	0.04 (0.04)	0.296	−0.11 (0.05) *	0.030	0.04 (0.06)	0.430
*Control Variables*												
Child Maltreatment (0–12 years)	0.03 (0.03)	0.322	−0.01 (0.04)	0.793	−0.02 (0.03)	0.654	0.01 (0.04)	0.748	0.00 (0.03)	0.972	−0.06 (0.04)	0.119
Female	−0.06 (0.03)	0.087	−0.02 (0.03)	0.591	−0.02 (0.03)	0.524	−0.02 (0.04)	0.544	0.08 (0.03) *	0.021	0.15 (0.04) ***	0.000
Black (ref.: White)	0.04 (0.05)	0.456	0.12 (0.05) **	0.008	0.25 (0.04) ***	0.000	0.09 (0.05)	0.079	0.06 (0.05)	0.186	−0.15 (0.05) **	0.002
Other (ref.: White)	−0.04 (0.05)	0.474	0.12 (0.04) **	0.007	0.12 (0.04) **	0.001	0.04 (0.05)	0.428	0.05 (0.05)	0.279	−0.11 (0.05) *	0.018
Poverty	−0.01 (0.04)	0.745	−0.00 (0.04)	0.987	0.00 (0.04)	0.914	−0.09 (0.04) *	0.031	−0.05 (0.04)	0.145	0.04 (0.04)	0.403
Outcome (16 years)	0.39 (0.04) ***	0.000	0.40 (0.03) ***	0.000	0.38 (0.03) ***	0.000	0.39 (0.04) ***	0.000	0.41 (0.04) ***	0.000	-	-

Notes: β = standardized beta, SE = standard error, B = unstandardized beta, ref. = reference. * *p* < 0.05, ** *p <* 0.01, *** *p <* 0.001.

**Table 3:** Fit Indices.

	χ^2^/Δχ^2^	RMSEA (90% CI)	TLI	SRMR
*Measurement Invariance Testing*				
Food Security				
Configural		0.063 (0.060, 0.065)	0.944	0.082
Metric	56.265 (42), *p* = 0.070	0.051 (0.049, 0.054)	0.962	0.084
Housing Stability				
Configural		0.059 (0.057, 0.061)	0.951	0.068
Metric	68.322 (42), *p = 0*.006	0.049 (0.046, 0.051)	0.966	0.072
*Structural Models*				
Single Group	4818.46 (1802), *p* < 0.001	0.041 (0.039, 0.042)	0.936	0.059
Multigroup Models for Food Security				
Healthcare	4245.03 (2366), *p* < 0.001	0.040 (0.038, 0.042)	0.963	0.071
Dental care	4251.46 (2366), *p* < 0.001	0.040 (0.038, 0.042)	0.963	0.071
Mental healthcare	4242.73 (2366), *p* < 0.001	0.040 (0.038, 0.042)	0.963	0.072
Self-rated health	4244,03 (2366), *p* < 0.001	0.040 (0.038, 0.042)	0.963	0.071
High School graduation/ enroll.	4261.42 (2366), *p* < 0.001	0.040 (0.038, 0.042)	0.963	0.072
Prosocial Activities	4341.50 (2447), *p* < 0.001	0.039 (0.037, 0.041)	0.963	0.072
Companionship	4323.87 (2549), *p* < 0.001	0.037 (0.035, 0.039)	0.965	0.071
Conflict with friends	4343.98 (2447), *p* < 0.001	0.039 (0.038, 0.041)	0.963	0.072
Satisfaction with friend	4171.64 (2445), *p* < 0.001	0.038 (0.036, 0.040)	0.967	0.069
Intimacy	4380.97 (2447), *p* < 0.001	0.040 (0.038, 0.042)	0.962	0.072
Independent Living Skills	4127.18 (2240), *p* < 0.001	0.041 (0.039, 0.043)	0.962	0.065
Multigroup Models for Housing Stability				
Healthcare	4309.47 (2366), *p* < 0.001	0.041 (0.039, 0.043)	0.961	0.073
Dental care	4308.49 (2366), *p* < 0.001	0.041 (0.039, 0.042)	0.961	0.073
Mental healthcare	4303.22 (2366), *p* < 0.001	0.041 (0.039, 0.042)	0.961	0.074
Self-rated health	4226.24 (2466), *p* < 0.001	0.038 (0.036, 0.040)	0.965	0.073
High School graduation/ enroll.	4313.71 (2366), *p* < 0.001	0.041 (0.039, 0.043)	0.961	0.073
Prosocial Activities	4400.82 (2447), *p* < 0.001	0.040 (0.038, 0.042)	0.961	0.072
Companionship	4378.14 (2549), *p* < 0.001	0.038 (0.036, 0.040)	0.964	0.073
Conflict with friends	4409.02 (2447), *p* < 0.001	0.040 (0.038, 0.042)	0.961	0.073
Satisfaction with friend	4392.32 (2447), *p* < 0.001	0.040 (0.038, 0.042)	0.961	0.072
Intimacy	4439.41 (2447), *p* < 0.001	0.040 (0.039, 0.042)	0.960	0.073
Independent Living Skills	4249.95 (2240), *p* < 0.001	0.042 (0.040, 0.044)	0.958	0.065

Notes: RMSEA = Root Mean Square Error of Approximation; TLI = Tucker Lewis Index; SRMR = Standardized Root Mean Squared Residual.

**Table 4:** Multigroup Structural Equation Models Predicting Functional Adaptations as Moderated by Food Security (*N* = 1003).

	Received Medical Check-Up		Received Dental Care		Received Needed Counseling/Therapy		Self-Rated Health		Graduated or in High School			
	FS B (SE)	FI B (SE)	FS B (SE)	FI B (SE)	FS B (SE)	FI B (SE)	FS B (SE)	FI B (SE)	FS B (SE)		FI B (SE)	
*Adolescent Neglect*												
IM	−0.21 (0.11)	−0.00 (0.14)	0.03 (0.13)	0.20 (0.14)	0.25 (0.18)	0.16 (0.23)	0.21 (0.10) *	0.19 (0.12)	−0.29 (0.21)		0.17 (0.16)	
IBN	−0.17 (0.32)	−0.29 (0.26)	−0.07 (0.41)	−0.90 (0.31) **	0.53 (0.64)	−0.42 (0.47)	−0.33 (0.26)	−0.15 (0.23)	0.80 (0.64)		−0.28 (0.34)	
PM	0.07 (0.14)	0.08 (0.13)	0.06 (0.16)	0.12 (0.14)	0.34 (0.23)	−0.10 (0.20)	0.14 (0.11)	0.08 (0.11)	−0.56 (0.30)		−0.14 (0.14)	
ERS	−0.12 (0.15)	0.18 (0.16)	−0.29 (0.18)	0.30 (0.16)	−0.32 (0.31)	0.16 (0.25)	0.18 (0.12)	0.06 (0.13)	0.11 (0.26)		0.005 (0.19)	
IS	−0.15 (0.24)	0.14 (0.19)	−0.03 (0.31)	0.29 (0.21)	−0.86 (0.47)	0.21 (0.32)	−0.30 (0.20)	−0.31 (0.18)	−0.41 (0.51)		0.00 (0.25)	
*Adolescent Abuse*												
PA	0.07 (0.12)	0.05 (0.10)	0.10 (0.16)	0.05 (0.08)	0.21 (0.32)	−0.22 (0.20)	−0.03 (0.06)	0.05 (0.08)	−0.00 (0.27)		0.14 (0.11)	
EA	−0.17 (0.12)	0.02 (0.08)	−0.11 (0.14)	0.01 (0.05)	−0.05 (0.14)	−0.06 (0.07)	−0.06 (0.04)	−0.07 (0.04)	0.05 (0.24)		0.02 (0.06)	
SA	0.31 (0.24)	−0.03 (0.07)	0.65 (0.55)	−0.04 (0.06)	0.11 (0.41)	0.24 (0.40)	0.22 (0.05) ***	−0.04 (0.06) ^1^	−0.12 (0.19)		−0.03 (0.06)	
*Control Variables*												
CM (0–12 years)	−0.03 (0.07)	−0.06 (0.07)	−0.25 (0.19)	−0.19 (0.17)	−1.06 (0.47) *	0.12 (0.28)	−0.22 (0.14)	−0.07 (0.14)	−0.13 (0.26)		−0.42 (0.20) *	
Female	0.17 (0.07) *	0.23 (0.07) ***	0.25 (0.16)	0.17 (0.14)	−0.48 (0.28)	−0.19 (0.20)	−0.42 (0.12) **	−0.46 (0.12) ***	0.39 (0.24)		0.21 (0.16)	
B (ref.: non-B)	−0.00 (0.07)	0.13 (0.08)	−0.20 (0.17)	−0.02 (15)	0.05 (0.23)	−0.04 (0.20)	0.00 (0.12)	0.15 (0.12)	0.50 (0.27)		−0.15 (0.17)	
Poverty	0.01 (0.07)	0.04 (0.07)	−0.49 (0.20) *	−0.39 (0.22)	−0.04 (0.27)	0.12 (0.28)	0.26 (0.14)	0.13 (0.18)	−0.84 (0.44)		−0.90 (0.43) *	
Outcome (16 years)												
	**Prosocial Activities**		**Companionship**		**Conflict**		**Satisfaction**		**Intimacy**		**Total Independent Living Skills**	
	**FS** **β (SE)**	**FI** **β (SE)**	**FS** **β (SE)**	**FI** **β (SE)**	**FS** **β (SE)**	**FI** **β (SE)**	**FS** **β (SE)**	**FI** **β (SE)**	**FS** **β (SE)**	**FI** **β (SE)**	**FS** **β (SE)**	**FI** **β (SE)**
*Adolescent Neglect*												
IM	0.01 (0.09)	−0.08 (0.08)	0.02 (0.08)	−0.27 (0.09) ** ^2^	−0.14 (0.08)	−0.01 (0.09)	−0.15 (0.08)	−0.10 (0.08)	−0.15 (0.07) *	−0.15 (0.09)	−0.10 (0.09)	−0.08 (0.09)
IBN	−0.18 (0.21)	0.04 (0.18)	0.00 (0.22)	0.03 (0.19)	−0.32 (0.25)	−0.07 (0.20)	−0.20 (0.24)	0.02 (0.17)	−0.19 (0.22)	0.22 (0.19)	0.10 (0.25)	0.26 (0.19)
PM	−0.07 (0.08)	−0.17 (0.08) *	0.02 (0.09)	0.04 (0.09)	0.10 (0.10)	−0.02 (0.09)	0.13 (0.09)	−0.09 (0.09)	0.04 (0.08)	−0.10 (0.09)	−0.04 (0.10)	−0.10 (0.09)
ERS	0.02 (0.11)	0.09 (0.10)	−0.07 (0.10)	−0.09 (0.11)	0.01 (0.12)	0.05 (0.12)	0.05 (0.12)	−0.06 (0.10)	0.06 (0.10)	−0.04 (0.12)	−0.20 (0.12)	−0.08 (0.10)
IS	0.15 (0.17)	−0.06 (0.13)	−0.07 (0.17)	0.06 (0.12)	0.43 (0.17) *	0.20 (0.15)	0.07 (0.16)	−0.03 (0.12)	0.09 (0.16)	−0.00 (0.12)	−0.04 (0.18)	−0.29 (0.14) *
*Adolescent Abuse*												
PA	−0.05 (0.04)	0.01 (0.05)	−0.01 (0.07)	0.05 (0.05)	0.06 (0.07)	−0.01 (0.05)	0.04 (0.08)	0.07 (0.04)	−0.03 (0.08)	0.05 (0.06)	−0.06 (0.05)	−0.10 (0.07)
EA	0.14 (0.06) *	−0.01 (0.05)	0.03 (0.09)	−0.06 (0.05)	−0.10 (0.10)	0.05 (0.05)	0.05 (0.09)	−0.07 (0.04)	0.06 (0.09)	−0.05 (0.06)	−0.09 (0.07)	0.12 (0.07)
SA	−0.04 (0.07)	−0.02 (0.06)	−0.07 (0.07)	−0.09 (0.04) *	0.11 (0.05) *	−0.05 (0.03) ^3^	−0.09 (0.04)	−0.01 (0.04)	−0.14 (0.07) *	−0.09 (0.06)	0.02 (0.06)	0.08 (0.11)
*Control Variables*												
CM (0–12 years)	0.02 (0.12)	0.00 (0.12)	−0.22 (0.12)	−0.07 (0.12)	−0.15 (0.11)	−0.01 (0.12)	0.01 (0.11)	−0.11 (0.13)	−0.04 (0.12)	−0.05 (0.13)	-	-
Female	−0.08 (0.10)	−0.06 (0.10)	−0.04 (0.10)	0.02 (0.10)	0.01 (0.10)	0.09 (0.10)	−0.10 (0.10)	0.29 (0.10) **	0.33 (0.10) ***	0.47 (0.09) ***	0.08 (0.05)	0.18 (0.05) ***
B (ref.: non-B)	0.01 (0.05)	0.12 (0.06) *	0.04 (0.05)	0.01 (0.05)	0.17 (0.11)	0.15 (0.06) *	0.03 (0.05)	0.04 (0.05) **	0.05 (0.06)	−0.04 (0.05)	−0.07 (0.05)	−0.03 (0.05)
Poverty	0.15 (0.11)	0.04 (0.19)	0.11 (0.11)	0.13 (0.15)	0.25 (0.12) *	0.34 (0.16) *	−0.28 (0.12) *	0.02 (0.18)	−0.10 (0.12)	−0.11 (0.14)	0.01 (0.06)	−0.01 (0.05)
Outcome (16 years)	−0.44 (0.04) ***	0.37 (0.05) ***	0.28 (0.04) ***	0.42 (0.04) ***	0.35 (0.05) ***	0.38 (0.05) ***	0.32 (0.04) ***	0.35 (0.04) ***	0.35 (0.05) ***	0.38 (0.04) ***		

Notes: Moderation is indicated by underline. Β = standardized, SE = standard error, B = unstandardized, ref. = reference. FS = food secure, FI = food insecure, IM = Inadequate Monitoring, IBN = Inattention to Basic Needs, PM = Permitting Misbehavior, ERS = Exposure to Risky Situations, IS = Inadequate Support, PA = physical abuse, EA = emotional abuse, SA = sexual abuse, CM = child maltreatment, B = Black. Outcome (16) signifies the prior level of the outcome variable, when available (e.g., prosocial activities at age 16). Due to nonconvergence, models were run for each outcome individually. Model adjustments included simplifying the race variable to Black or non-Black. The childhood maltreatment variable was trimmed from the independent living model to resolve non-convergence. ^1^ Difference = 0.26 (0.08) *p* = 0.001. *^2^* Difference *=* 0.20 (0.08), *p* = 0.013; ^3^ Difference = −0.12 (0.05), *p* = 0.014. * *p* < 0.05, ** *p <* 0.01, *** *p <* 0.001.

**Table 5:** Multigroup Structural Equation Models Predicting Functional Adaptations as Moderated by Housing Stability (*N* = 1003).

	Received Medical Check-Up		Received Dental Care		Received Needed Counseling/Therapy		Self-Rated Health		Graduated or Enrolled in High School			
	SH B (SE)	UH B (SE)	SH B (SE)	UH B (SE)	SH B (SE)	UH B (SE)	SH B (SE)	UH B (SE)	SH B (SE)		UH B (SE)	
*Adolescent Neglect*												
IM	0.07 (0.11)	0.21 (0.20)	0.05 (0.10)	0.02 (0.17)	0.12 (0.13)	0.23 (0.32)	0.17 (0.09)	0.34 (0.15) *	−0.32 (0.16) *		0.52 (0.22) * ^4^	
IBN	−0.29 (0.28)	−0.09 (0.42)	−0.32 (0.25)	−0.83 (0.41) *	0.06 (0.42)	−0.94 (0.89)	−0.13 (0.22)	−0.37 (0.29)	−0.66 (0.42)		0.91 (0.60)	
PM	0.13 (0.14)	0.09 (0.17)	0.12 (0.13)	0.06 (0.13)	0.16 (0.19)	0.16 (0.23)	0.05 (0.10)	0.16 (0.11)	−0.12 (0.17)		−0.41 (0.20) *	
ERS	0.06 (0.14)	−0.05 (0.24)	−0.09 (0.13)	0.36 (0.22)	−0.24 (0.22)	0.66 (0.44)	0.08 (0.11)	0.19 (0.16)	0.33 (0.20)		−0.46 (0.31)	
IS	0.05 (0.20)	−0.17 (0.34)	0.03 (0.19)	0.46 (0.31)	−0.23 (0.29)	0.17 (0.55)	−0.32 (0.18)	−0.35 (0.23)	0.62 (0.33)		−10.02 (0.47) * ^5^	
*Adolescent Abuse*												
PA	0.09 (0.12)	−0.07 (0.22)	0.13 (0.10)	−0.04 (0.09)	−0.05 (0.10)	−0.27 (0.31)	−0.00 (0.07)	0.07 (0.07)	0.38 (0.20)		−0.08 (0.15)	
EA	−0.00 (0.06)	−0.07 (0.10)	0.04 (0.06)	−0.05 (0.05)	−0.10 (0.06)	−0.04 (0.15)	−0.07 (0.04)	−0.05 (0.04)	−0.11 (0.12)		0.16 (0.11)	
SA	−0.06 (0.07)	0.35 (0.42)	0.01 (0.05)	0.06 (0.07)	0.06 (0.31)	0.33 (0.49)	−0.01 (0.05)	0.02 (0.06)	−0.17 (0.11)		−0.00 (0.09)	
*Control Variables*												
CM (0-12 years)	−0.07 (0.15)	−0.17 (0.27)	−0.16 (0.13)	−0.39 (0.24)	−0.15 (0.20)	−0.76 (0.58)	−0.18 (0.12)	−0.05 (0.19)	−0.32 (0.20)		−0.23 (0.29)	
Female	0.31 (0.13) *	0.62 (0.23) **	0.28 (0.12) *	0.05 (0.16)	−0.51 (0.20) *	0.01 (0.28)	−0.39 (0.10) ***	−0.50 (0.14) **	0.51 (0.19) **		0.11 (0.21)	
B (ref.: non-B)	0.11 (0.14)	0.16 (0.19)	−0.07 (0.13)	−0.08 (0.17)	−0.14 (0.17)	0.26 (0.26)	−0.03 (0.11)	0.24 (0.13)	−0.02 (0.22)		0.10 (0.21)	
Poverty	0.13 (0.15)	−0.09 (0.29)	−0.44 (0.15) *	−0.37 (0.25)	−0.21 (0.24)	0.40 (0.38)	0.09 (0.12)	0.40 (0.22)	−0.78 (0.36) *		−10.28 (0.60) *	
Outcome (16 years)												
	**Prosocial Activities**		**Companionship**		**Conflict**		**Satisfaction**		**Intimacy**		**Total Independent Living Skills**	
	**SH** **β (SE)**	**US** **β (SE)**	**SH** **β (SE)**	**US** **β (SE)**	**SH** **β (SE)**	**UH** **β (SE)**	**SH** **β (SE)**	**UH** **β (SE)**	**SH** **β (SE)**	**UH** **β (SE)**	**SH** **β (SE)**	**UH** **β (SE)**
*Adolescent Neglect*												
IM	−0.16 (0.07) *	0.17 (0.12) ^6^	−0.07 (0.07)	−0.24 (0.11) *	−0.11 (0.06)	−0.04 (0.13)	−0.08 (0.06)	−0.19 (0.10)	−0.09 (0.07)	−0.22 (0.10) *	−0.08 (0.07)	−0.04 (0.12)
IBN	0.01 (0.18)	−0.15 (0.23)	−0.06 (0.18)	−0.13 (0.23)	−0.10 (0.18)	−0.11 (0.29)	−0.22 (0.18)	0.00 (0.25)	−0.19 (0.18)	0.38 (0.23)	0.06 (0.19)	0.44 (0.27)
PM	−0.16 (0.09)	−0.14 (0.08)	0.01 (0.09)	0.06 (0.10)	0.03 (0.08)	0.05 (0.11)	0.03 (0.08)	−0.01 (0.09)	−0.01 (0.08)	−0.08 (0.09)	0.06 (0.08)	−0.24 (0.11) ^7^
ERS	−0.04 (0.09)	0.16 (0.12)	−0.15 (0.09)	0.22 (0.13)	0.02 (0.10)	−0.01 (0.17)	0.09 (0.09)	−0.13 (0.13)	0.13 (0.10)	−0.19 (0.13)	−0.16 (0.10)	−0.17 (0.13)
IS	0.11 (0.14)	−0.06 (0.19)	0.02 (0.14)	0.10 (0.17)	0.28 (0.13) *	0.21 (0.22)	0.02 (0.13)	0.13 (0.19)	0.11 (0.13)	−0.13 (0.18)	−0.16 (0.14)	−0.32 (0.21)
*Adolescent Abuse*												
PA	0.02 (0.05)	−0.02 (0.05)	0.07 (0.05)	−0.08 (0.07)	0.01 (0.06)	0.07 (0.06)	0.05 (0.04)	0.10 (0.08)	0.07 (0.05)	−0.03 (0.10)	−0.12 (0.04) **	−0.02 (0.10)
EA	−0.06 (0.05)	0.16 (0.07) * ^8^	−0.06 (0.06)	0.02 (0.07)	0.03 (0.06)	−0.03 (0.08)	−0.09 (0.05)	−0.00 (0.06)	−0.04 (0.06)	0.07 (0.08)	0.06 (0.06)	−0.07 (0.09)
SA	−0.05 (0.07)	−0.05 (0.08)	−0.01 (0.05)	−0.13 (0.06) *	−0.01 (0.04)	−0.02 (0.05)	−0.03 (0.03)	−0.06 (0.06)	−0.02 (0.05)	−0.17 (0.08) *	0.07 (0.06)	−0.03 (0.11)
*Control Variables*												
CM (0–12 years)	0.09 (0.10)	−0.14 (0.16)	−0.14 (0.11)	−0.14 (0.16)	−0.11 (0.10)	0.02 (0.16)	−0.09 (0.10)	0.10 (0.16)	0.04 (0.10)	−0.22 (0.18)		
Female	−0.02 (0.09)	0.03 (0.12)	−0.11 (0.09)	−0.20 (0.11)	0.04 (0.09)	0.06 (0.12)	0.16 (0.09)	0.01 (0.12)	0.45 (0.08) ***	0.31 (0.11) **	0.16 (0.04) ***	0.09 (0.06)
B (ref.: non-B)	0.10 (0.06)	0.03 (0.06)	0.02 (0.04)	0.03 (0.06)	0.13 (0.06) ^*^	0.22 (0.14)	0.09 (0.06)	−0.03 (0.06)	0.04 (0.04)	−0.05 (0.06)	−0.02 (0.05)	−0.08 (0.06)
Poverty	−0.07 (0.11)	−0.01 (0.19)	0.05 (0.10)	0.36 (0.18) *	0.32 (0.11) **	0.26 (0.18)	−0.21 (0.12)	−0.08 (0.19)	−0.03 (0.11)	−0.17 (0.18)	−0.03 (0.05)	0.10 (0.06)
Outcome (16 years)	0.39 (0.04) ***	0.41 (0.05) ***	0.36 (0.04) ***	0.32 (0.05) ***	0.40 (0.04) ***	0.29 (0.08) ***	.40 (0.04) ***	0.28 (0.05) ***	0.40 (0.04) ***	0.37 (0.05) ***		

Notes: Moderation is indicated by underline. Β = standardized, SE = standard error, B = unstandardized, ref. = reference. SH = stable housing, US = unstable housing, IM = Inadequate Monitoring, IBN = Inattention to Basic Needs, PM = Permitting Misbehavior, ERS = Exposure to Risky Situations, IS = Inadequate Support, PA = physical abuse, EA = emotional abuse, SA = sexual abuse, CM = child maltreatment, B = Black. Outcome (16) signifies the prior level of the outcome variable, when available (e.g., prosocial activities at age 16). Due to nonconvergence, models were run for each outcome individually. Model adjustments included omitting item 20 from IBN, removing the error covariance between items 1 and 2, and simplifying the race variable to Black or non-Black. Childhood maltreatment was trimmed from the independent living model to resolve non-convergence. ^4^ Difference = 0.85 (0.28), *p* = 0.002; ^5^ Difference = 1.65 (0.57), *p* =0.004. ^6^ Difference = −0.64 (0.26), *p* = 0.013; ^7^ Difference = 3.77 (1.77), *p* = 0.033; ^8^ Difference = −0.25 (0.10). *p* = 0.014. * *p* < 0.05, ** *p <* 0.01, *** *p <* 0.001.

## Data Availability

This publication utilizes data from the Longitudinal Studies of Child Abuse and Neglect, which have been provided by the National Data Archive on Child Abuse and Neglect (NDACAN), a service of the Children’s Bureau, U.S. Department of Health and Human Services. Nothing herein should be construed to indicate the support or endorsement of its content by the collector of the original data, their funding agency, NDACAN, or ACF/DHHS.

## Supplementary Materials

The following supporting information can be downloaded at: https://www.mdpi.com/article/10.3390/children9030390/s1. Figure S1: Measurement model for adolescent neglect.
