# Supplementary figures and images for: Neglect, Abuse, and Adaptive Functioning: Food Security and Housing Stability as Protective Factors for Adolescents
Source: Children (Basel). 2022 Mar 10;9(3):390. doi: 10.3390/children9030390 (PMC8946869; doi:10.3390/children9030390)

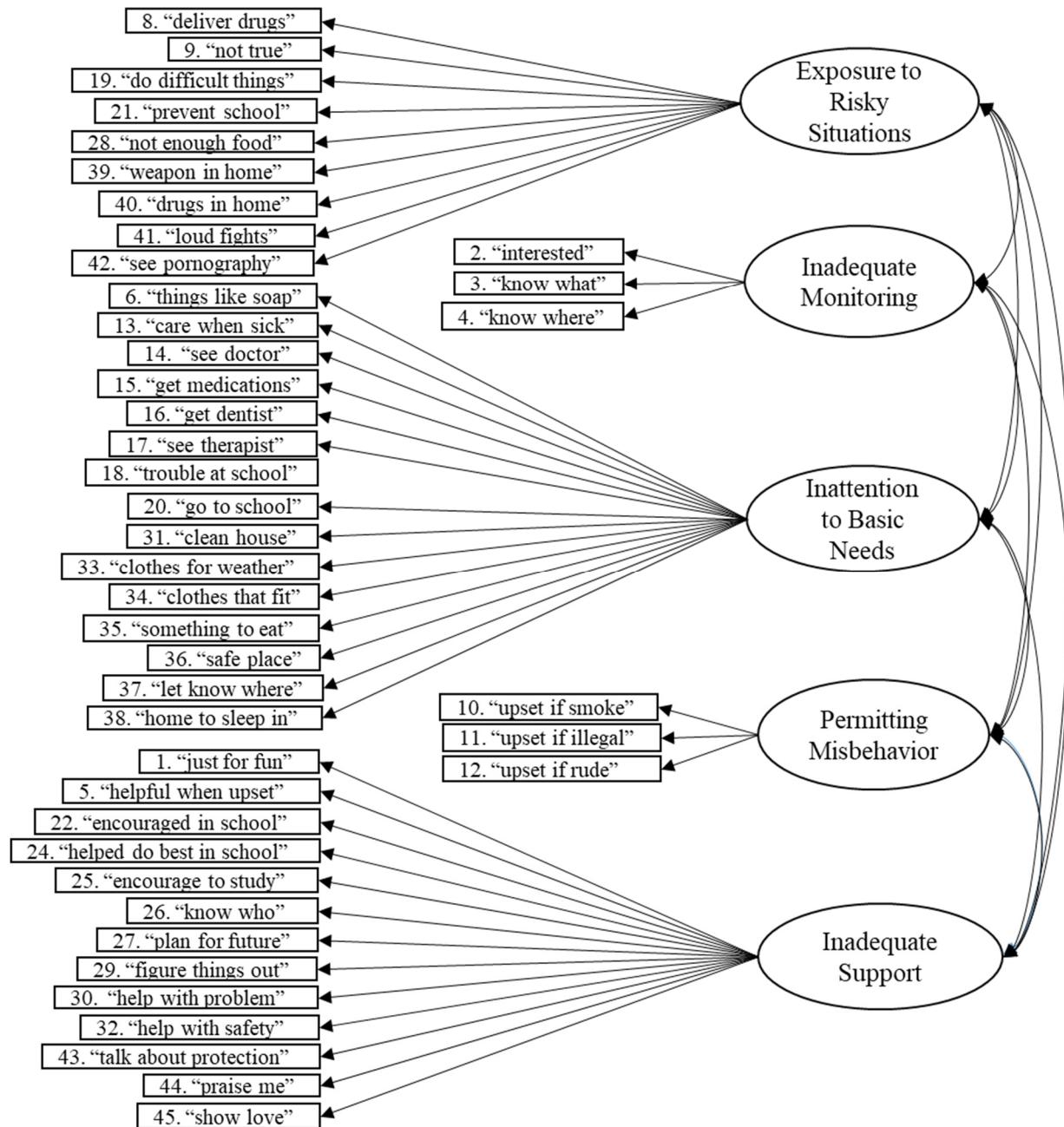

Figure S1: Measurement Model for Mid-adolescent Neglect

Supplement: Supplementary file 1 [file children-09-00390-s001.zip › children-1547575-supplementary.pdf]
